# Association of Common Variants in *TCF4* and *PTPRG* with Fuchs' Corneal Dystrophy: A Systematic Review and Meta-Analysis

**DOI:** 10.1371/journal.pone.0109142

**Published:** 2014-10-09

**Authors:** Lawrence C. M. Lau, Li Ma, Alvin L. Young, Shi Song Rong, Vishal Jhanji, Marten E. Brelen, Chi Pui Pang, Li Jia Chen

**Affiliations:** 1 Department of Ophthalmology and Visual Sciences, Prince of Wales hospital, Hong Kong, China; 2 Department of Ophthalmology and Visual Sciences, the Chinese University of Hong Kong, Hong Kong, China; University of Utah, United States of America

## Abstract

**Topic:**

A meta-analysis of *TCF4* and *PTPRG* gene variants in Fuchs' corneal dystrophy (FCD).

**Clinical relevance:**

To identify novel genetic markers in patients with FCD in different ethnic populations.

**Methods:**

MEDLINE and EMBASE were searched for eligible genetic studies on *TCF4* and *PTPRG* in FCD. Odds ratios (OR) and 95% confidence intervals (CI) of each single-nucleotide polymorphism (SNP) in allelic, dominant and recessive models were estimated using fixed-effect model if *I^2^*<50% in the test for heterogeneity, otherwise the random effects model was used.

**Results:**

Thirty-three records were obtained, with 8 being suitable for meta-analysis, which included five SNPs in *TCF4* and two in *PTPRG*. There were 1610 FCD patients and 1565 controls tested for *TCF4* rs613872. This SNP was strongly associated with FCD in Caucasians (P = 5.0×10^−106^), with the risk allele G conferring an OR of 3.95 (95% CI: 3.49–4.46). A further 4 *TCF4* SNPs (rs17595731, rs2286812, rs618869 and rs9954153) were also significantly associated with FCD in Caucasians (P<10^−8^). However, we found no SNP associated with FCD in Chinese. In addition, there was no significant association between FCD and *PTPRG*.

**Conclusion:**

*TCF4* rs613872 is strongly associated with FCD in Caucasians but not in Chinese, which may suggest ethnic diversity in FCD SNP associations. SNPs in *PTPRG* were not associated with FCD in Caucasians or Chinese populations. Results of this meta-analysis indicate the need for larger-scale and multi-ethnic genetic studies on FCD to further explore the associated gene variants and their roles on the mechanism and genetic basis of FCD.

## Introduction

Fuchs' corneal dystrophy (FCD) (MIM 136800) is a bilateral, asymmetric, progressive disorder affecting the corneal endothelium. It is characterized by the formation of guttata which are microscopic outgrowths on Descemet membrane. The resulting thickening of Descemet membrane causes a loss of corneal endothelial cells leading to corneal edema and eventual visual loss [1]–[4]. FCD affects 4% of the United States population aged 40 years and above and is a leading indication for corneal transplantation [1]–[4].

The etiology of FCD is not fully understood. Genetic factors have been suggested as a major risk factor [1], [5]. Four FCD loci have been identified by genetic linkage analysis, namely *FCD1, FCD2, FCD3* and *FCD4* on chromosome 13, 18, 5, and 9 respectively [6]–[11]. *ZEB1* located on 10p11.2, *SLC4A11* on 20p12 and *LOXHD1* on 18q21.1 have been reported as causal genes [5]. *ZEB1*, encoding the Zinc finger E-box-binding homeobox 1 transcription factor, also known as *TCF8*, harbors the missense mutations p.Q840P, p.N78T, p.P649A, p.Q810P and p.A905T in patients with FCD [10], [12]. There were 11 mutations in *SLC4A11* that have been identified in both sporadic and familial cases [10], [13]. Depletion of SLC4A11 in cultured human corneal endothelial cells resulted in the reduction of cellular proliferation with increased apoptosis [14]. *LOXHD1* is located on chromosome 18 (FCD2) and mutations of LOXHD1 result in aggregates in the endothelium and increased thickness with protein abundance of the Descemet membrane which are both pathognomonic of FCD [15]. A nonsense mutation in *AGBL1* in the 15q locus has been identified in patients with FCD recently and its truncated protein product AGBL1 has altered protein-protein interaction with protein transcription factor 4 (TCF4) [16]. Another rare early-onset form of FCD has been linked to *COL8A2* on chromosome 1p34.3-p32.3. This type of FCD shows gender differences but still has histopathological characteristics of the endothelial guttata from the classic late-onset form of FCD. It has therefore been regarded as a different form of the same disease [5].

In 2010 a genome-wide association study (GWAS) identified the single-nucleotide polymorphisms (SNPs) rs613872 in *TCF4* and rs10490775 in the *protein tyrosine phosphatase receptor type G* (*PTPRG*) gene in FCD patients [17]. A strong association of rs10490775 was found but did not reach genome-wide statistical significance in the combined analysis. Replication studies supported the association of rs613872 with FCD in Caucasians [18]–[22] but not in Chinese [23], [24]. Other SNPs such as rs17089925 and rs17089887 were reported to be associated in Chinese [24]. The *TCF4* gene is located on chromosomal region 18q21. The upstream gene, *CCDC68*, is over 260 kbps away from *TCF4*, while the downstream hypothetical gene, *FLJ45743*, is over 600 kbps away (HapMap Data Release 28). Therefore, rs613872 is less likely to tag another gene in this region. The *TCF4* gene encodes the E2-2 protein, which is expressed in the developing corneal epithelium [17]. *TCF4* can induce epithelial-mesenchymal transition (EMT) in epithelial cells and is vital in corneal damage repair [5], [17]. *TCF4* rs613872 is within binding site for two transcription factors; *Ini1 (SMARCB1)* and *BRG1 (SMARCA4)* which are components of the SWI/SNF chromatin remodeling complex involved in transcriptional regulation [25], [26]. Although according to GWAS the *TCF4* and *PTPRG* genes are significantly associated with FCD, these genes also show heterogeneity in the association profiles across populations. Thus the current meta-analysis will aim to give a comprehensive review of all the relevant studies to demonstrate clarity in association of these genes with FCD in different population groups. This is to establish the significance of *TCF4* and *PTPRG* related to FCD, with the highlight of relevant SNPs. Included in this meta-analysis are all the reported associations of SNPs in or near *TCF4* and *PTPRG* with FCD. The association credibility and the effects of the various genes are also evaluated.

## Methods

### Literature search

A systematic literature search was conducted in the MEDLINE and EMBASE databases (accessed July 30, 2013) with the following free words and MeSH terms: “E2-2”, “transcription factor 4”, “TCF-4”, “TCF4”, “protein tyrosine phosphatase, receptor type, G”, “HPTPG”, “PTPG”, “PTPRG” and “Corneal Dystrophies, Hereditary”, and “Fuchs' Endothelial Dystrophy”. We supplemented our search by screening the reference lists of all the relevant studies, including original articles, reviews, and meta-analyses. No language filter was applied.

### Inclusion and exclusion criteria

The following inclusion criteria were applied in the review process: (1) association studies from peer-reviewed journal evaluating the association between variants in selected genes and the disease; (2) genotype or allele counts and/or frequencies in case and control groups were presented, or such data could be calculated from enumerative data provided in the articles; (3) case and control groups were unrelated and drawn from the same temporally and geographically defined population; (4) control subjects are free of any form of corneal dystrophy. All animal studies, case reports, reviews, abstracts, conference proceedings, editorials and reports with incomplete data were excluded. Although non-English articles were not deliberately excluded, the articles in the final analysis were all in English. We also searched some Chinese databases (CBM, CNKI, VIP) but did not find any additional relevant studies. For studies published by the same group on the same gene and markers on overlapping sample population only the most recent article or the article with the largest sample size was included for analysis. Data from non-overlapped sample populations from same study are regarded as different sample collections.

### Literature review and data extraction

Two reviewers (LL and LM) separately extracted data from the retrieved records and confirmed the validity of the included articles. Any discrepancy was resolved by other reviewers (SSR and LJC). The following variables were extracted: author, year of publication, ethnicity of study subjects, result of Hardy-Weinberg equilibrium (HWE) test in controls, the numbers of patients and controls, demographic information, and the allele and genotype counts or frequencies of each SNP. The allele or genotype counts were calculated from the frequencies, rounding to the closest integer, in those studies where the counts were not given [20]–[22], [24].

### Meta-analysis and test for potential bias

Polymorphisms reported in two or more studies were meta-analyzed. Odds ratios (ORs) and 95% confidence interval (CIs) for the tested allele (or minor allele) were calculated with fixed effects model if *I^2^*≤50% or random effects model when *I^2^*>50% [27]. We also conducted subgroup analysis based on ethnicity where applicable. The results of individual studies were pooled using the software Review Manager (RevMan, version 5.2, The Cochrane Collaboration, Copenhagen, Denmark).

Inter-study heterogeneity was tested with the *I^2^* test. The *I^2^* value was interpreted as of no (0–25%), low (25–50%), moderate (50–75%) and high heterogeneity (75–100%) [28]. Statistical significance of the association between FCD and the polymorphism was evaluated with the *Z*-test. The P values were calculated using the Z scores. An association giving a pooled P value of <0.05 was considered statistically significant. The Funnel plots and Egger's test were used to evaluate potential biases [29], [30]. When the Egger test reported *P*<0.05, publication bias was assumed to exist.

## Results

### Characteristics of the included studies

**Figure 1:**
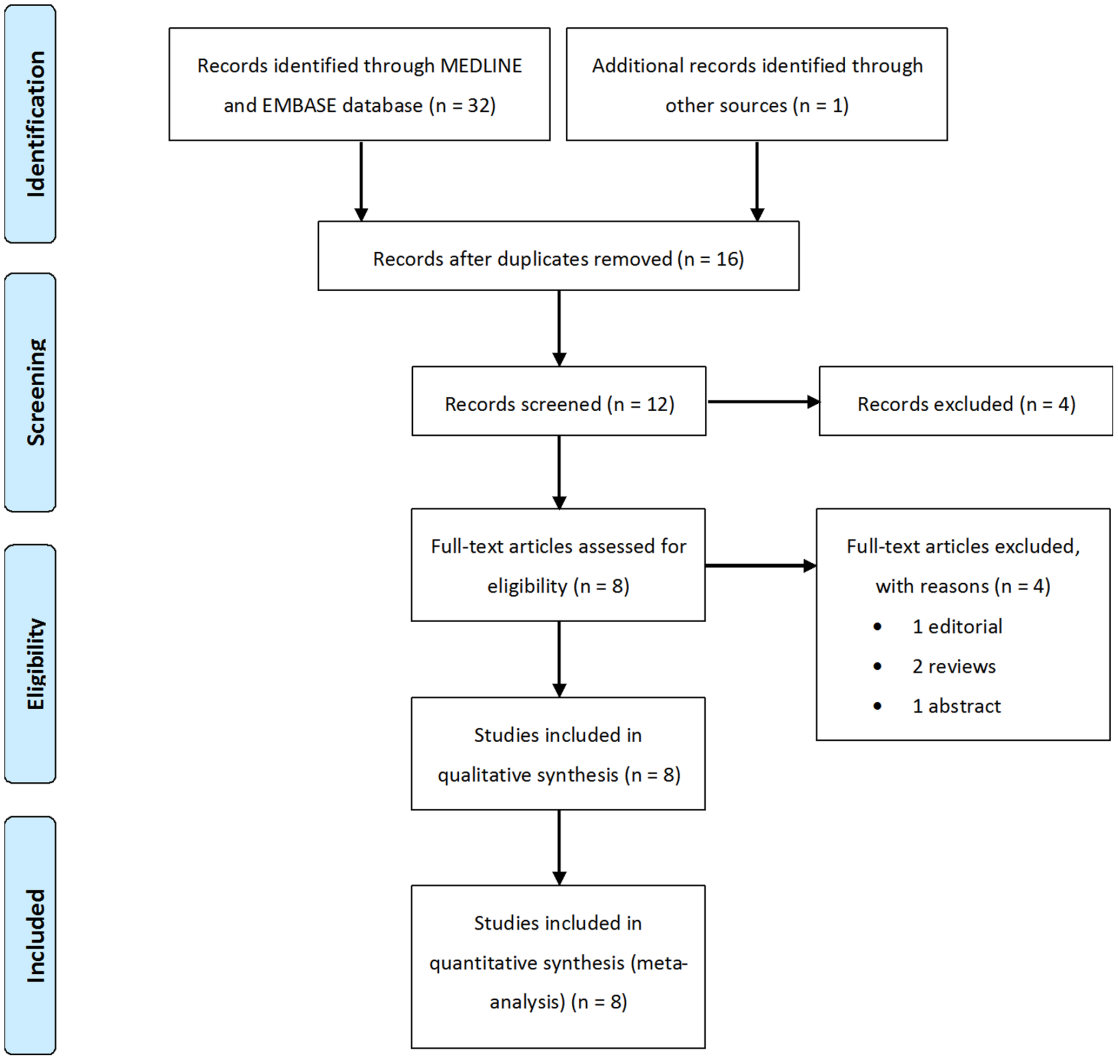
Flow diagram (modified from The PRISMA Flow Diagram) and results of literature review. Flow diagram depicted the screening process of retrieved articles, including the number and reason of exclusion.

The workflow and results of the literature review are shown in Figure 1. A total of 33 records representing 16 independent studies were identified from the search. Twelve of these met our study criteria; however, 4 were excluded since they were review papers or abstracts (Appendix S2). Therefore, 8 articles involving 9 sample collections were included in the final meta-analysis (Appendix S1).

The characteristics of all the included articles are summarized in Table 1. The 8 eligible studies included a total of 1,707 FCD cases and 2,184 controls [17]–[24]. Five SNPs (rs613872, rs17595731, rs2286812, rs618869 and rs9954153) in *TCF4* and 2 SNPs (rs7640737 and rs10490775) in *PTPRG* were tested in at least two sample collections and thus were eligible for meta-analysis.

**Table 1:** Characteristics of the studies included in the meta-analysis.

		Sample size		Gender (M/F)		Age, mean±SD, years		
Study	ethnicity	Cases	Controls	Cases	Controls	Cases	Controls	HWE
Baratz 2010a	Caucasian	130	260	30/100	60/200	77±9	77±9	YES
Baratz 2010b	Caucasian	150	150	46/104	46/104	74±8	74±8	YES
Thalamuthu 2011	Chinese	57	121	12/45	51/70	67	65.1	YES
Riazuddin 2011	Caucasian	170	180	62/108	82/98	66.25±12.97	71.99±7.52	YES
Li 2011	Caucasian	450	340	NA	NA	NA	NA	YES
Igo 2012	Caucasian	531	204	154/377	50/154	67±12	67±10	NA
Kuot 2012	Caucasian	103	275	35/68	137/138	68.6±15.5	76.0±8.4	YES
Wang 2013	Chinese	34	491	NA	219/272	NA	71.7±8.2	YES
Stamler 2013	Caucasian	82	163	NA	NA	NA	NA	YES

NA: not available. SD: standard deviation. HWE: Hardy-Weinberg equilibrium. Baratz 2010a and Baratz 2010b are from the same study but of different cohorts.

### Meta-analysis of *TCF4* and *PTPRG* polymorphisms

Five SNPs in the *TCF4* gene, namely rs613872, rs17595731, rs2286812, rs618869 and rs9954153, were meta-analyzed. Notably rs613872 and rs17595731 were non-polymorphic in Chinese [23], [24], thus the odds ratios could not be estimated for the Chinese population.

**Figure 2:**
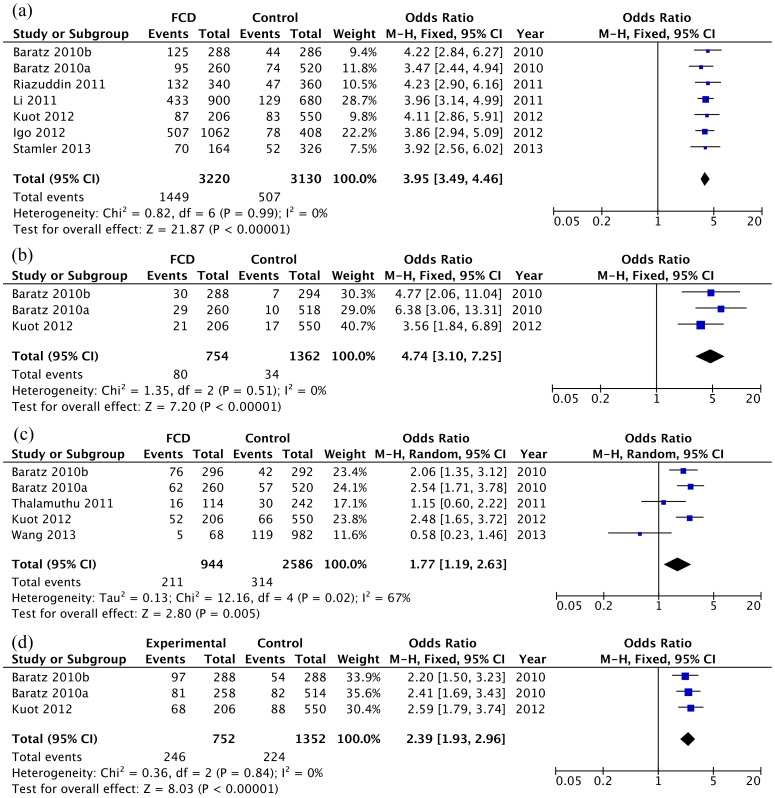
Forest plot of *TCF4* allelic model: (a) *TCF4* rs613872 (b) *TCF4* rs17595731 (c) *TCF4* rs2286812 (d) *TCF4* rs9954153. Squares indicate the study-specific odds ratio (OR). The size of the box is proportional to the weight of the study. Horizontal lines indicate 95% confidence interval (CI). A diamond indicates the summary OR with its corresponding 95% CI.

SNP rs613872 had been investigated in 9 study cohorts. Since it is not detected in Chinese, we only present data from Caucasians, consisting of 1610 FCD cases and 1565 controls. The allele G of rs613872 was more frequent in FCD patients than in controls in all Caucasian cohorts. It is strongly associated with FCD, conferring a pooled odds ratio of 3.95 (95% CI: 3.49–4.46, Z = 21.87, P = 5.0×10^−106^, *I^2^* = 0%; Figure 2a and Table 2). Rs17595731 was tested in 3 study cohorts totaling 377 cases and 681 controls. The pooled OR for the C allele was 4.74 (95% CI: 3.10–7.25, Z = 7.20, P = 6.0×10^−13^, *I^2^* = 0%; Figure 2b and Table 2). SNP rs2286812 was investigated in four studies with 472 cases and 1293 controls, comprising of both Chinese and Caucasians. The association was significant after pooling the data (OR = 1.77, 95% CI: 1.19–2.63, P = 0.00051, Figure 2c and Table 2); however, large heterogeneity (*I^2^* = 67%) was detected, which could be explained by the differences in Caucasians and Chinese cohorts. In Chinese, this SNP was not significantly associated with FCD (pooled OR = 0.88, 95% CI: 0.45–1.72, Z = 0.36, P = 0.72, *I^2^* = 31%). When the data from the two Chinese cohorts [23], [24] were removed, the association became strongly significant in Caucasians (OR = 2.36, 95% CI: 1.86–2.98, Z = 7.16, P = 8.1×10^−13^, *I^2^* = 0%). SNP rs618869 was tested in two cohorts from the same study by Baratz et al. [17] The T allele was significantly associated with FCD, with a pooled OR of 2.94 (95% CI: 2.23–3.89, Z = 7.60, P = 3.0×10^−14^, *I^2^* = 0%; Table 2). These two cohorts were separate sample collections [17] and should have low risk of overlapping subjects. SNP rs9954153 was tested in three cohorts from two studies and the G allele was significantly associated with FCD, with a pooled OR of 2.39 (95% CI: 1.93–2.96, Z = 8.03, P = 9.7×10^−16^, *I^2^* = 0%; Figure 2d and Table 2). In the dominant and recessive models, all SNPs showed a significant association with FCD except for *TCF4* rs17595731 in the recessive model (Table 2). This is likely due to the small pooled-sample size.

**Table 2:** Pooled analyses on the relationship of gene polymorphisms with FCD.

Polymorphism	Ethnicity	Allele	Number of cohorts	Sample size (Case/Control)	Genetic model	OR (95% CI)	Z score	P-value	I^2^ (%)	Reference
*TCF4* rs613872	Caucasian	G/T	7	1610/1565	G vs. T	3.95 (3.49–4.46)	21.87	5.0×10^−106^	0	[16]–[21]
					GG+GT vs. TT	6.05 (5.14–7.10)	21.84	9.7×10^−106^	12	
					GG vs. TT+GT	6.47 (4.55–9.20)	10.41	2.2×10^−25^	8	
*TCF4* rs17595731	Caucasian	C/G	3	377/681	C vs. G	4.74 [3.10–7.25]	7.20	6.0×10^−13^	0	[16], [20]
					CC+CG vs. GG	5.12 (3.29–7.96)	7.25	4.2×10^−13^	0	
					CC vs. GG+CG	5.20 (0.81–33.42)	1.74	0.082	0	
*TCF4* rs2286812	All ancestries	T/C	5	472/1293	T vs. C	1.77 (1.19–2.63)	2.80	0.0051	67	[16], [20], [22], [23]
					TT+TC vs. CC	1.87 (1.15–3.04)	2.53	0.011	70	
					TT vs. CC+TC	2.58 (1.29–5.14)	2.69	0.0071	0	
*TCF4* rs618869	Caucasian	T/C	2	276/403	T vs. C	2.94 (2.23–3.89)	7.60	3.0×10^−14^	0	[16]
					TT+TC vs. CC	3.77 (2.70–5.26)	7.81	5.7×10^−15^	0	
					TT vs. CC+TC	4.54 (1.69–12.18)	3.0	0.0027	44	
*TCF4* rs9954153	Caucasian	G/T	3	376/676	G vs. T	2.39 (1.93–2.96)	8.03	9.7×10^−16^	0	[16], [20]
					GG+GT vs. TT	2.96 (2.27–3.86)	7.99	1.3×10^−15^	0	
					GG vs. GT+TT	3.59 (1.90–6.80)	3.94	8.1×10^−5^	0	
*PTPRG* rs7640737	All ancestries	T/C	4	416/1175	T vs. C	1.56 (0.84–2.89)	1.42	0.16	83	[16], [20], [22]
					TT+TC vs. CC	1.54 (0.80–2.97)	1.30	0.19	81	
					TT vs. CC+TC	3.19 (0.68–14.93)	1.47	0.14	52	
*PTPRG* rs10490775	Caucasian	A/G	3	380/683	A vs. G	1.49 (0.67–3.27)	0.98	0.33	89	[16], [20]
					AA+AG vs. GG	1.45 (0.63–3.35)	0.88	0.38	87	
					AA vs. GG+AG	3.05 (0.35–26.35)	1.01	0.31	68	

**Figure 3:**
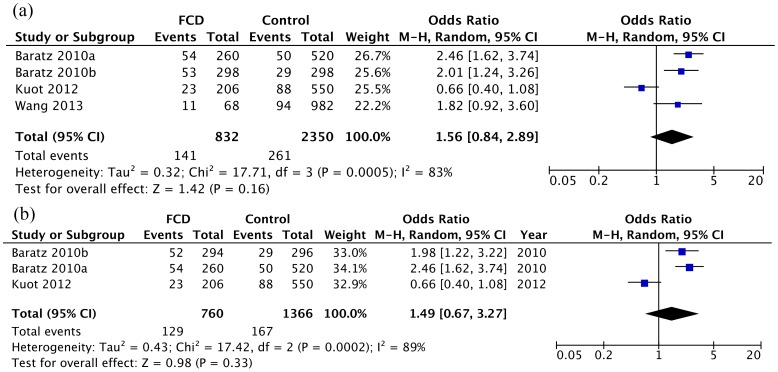
Forest plot of *PTPRG* allelic model: (a) *PTPRG* rs7640737 (b) *PTPRG* rs10490775. Squares indicate the study-specific odds ratio (OR). The size of the box is proportional to the weight of the study. Horizontal lines indicate 95% confidence interval (CI). A diamond indicates the summary OR with its corresponding 95% CI.

Two SNPs of the *PTPRG* gene were meta-analyzed. The rs7640737 has been studied in 4 sample collections totaling 416 FCD patients and 1175 control subjects. The minor allele T showed an opposite trend in the study of Kuot et al. as compared to the studies of Baratz et al. and Wang et al. The pooled OR was 1.56 (95% CI: 0.84–2.89, P = 0.16, *I^2^* = 83%; Figure 3a and Table 2), but not statistically significant. Similarly, the A allele of rs10490775 also showed opposite effects between the studies of Kuot et al. and Baratz et al., and it was not significantly associated with FCD (OR = 1.49, 95% CI: 0.67–3.27, P = 0.33, *I^2^* = 89%; Figure 3b and Table 2). These Two SNPs were not significantly associated with FCD in either the dominant or recessive models (Table 2).

### Test for potential biases

A funnel plot revealed a symmetric inverted shape and no significant bias was detected.

## Discussion

In a recent genome-wide association study Baratz et al. identified the SNP rs613872 in the *TCF4* gene to be significantly associated with FCD. They also found another 3 independently associated FCD SNPs (rs17595731, rs9954153 and rs2286812). Although the associations of FCD with SNPs in the *PTPRG* gene were indicated, the overall P value did not reach genome-wide significance [17]. These gene variants have subsequently been investigated in different ethnic populations however, the results were inconsistent and variable. In this systematic review and meta-analysis, a strong association of *TCF4* rs613872 with FCD in Caucasians (P = 5×10^−106^) was obtained and there was no heterogeneity found (*I^2^* = 0%). The G allele increases the odds of FCD by nearly 4 folds. In contrast, rs613872 is not associated with FCD in ethnic Chinese [23], [24]. In addition to rs613872, our data also revealed significant association of another 4 *TCF4* SNPs (rs17595731, rs2286812, rs618869 and rs9954153) with FCD in Caucasians (P<10^−8^). Rs2286812 has been studied in two Chinese cohorts but showed no significant association [23], [24]. In contrast to *TCF4*, SNPs rs10490775 and rs7640737 in *PTPRG* were not associated with FCD in Caucasians (P>0.1) and has distinct heterogeneity across study populations (*I^2^*>80%). Accordingly, *TCF4* is the only susceptibility gene that has been confirmed so far for FCD.

This meta-analysis involves the largest sample size to date with a total of 1610 FCD cases and 1565 controls. The presence of *TCF4* rs613872 in FCD among the Caucasian populations is confirmed and the G allele is a risk factor. However, *TCF4* rs613872 and rs17595731 has been investigated in a total of 91 FCD patients, 42 patients with non-Fuchs' corneal dystrophies, and 612 controls of Chinese origin [23], [24], but they were found to be non-polymorphic. Instead, another *TCF4* SNP rs17089887, near rs613872, was significantly associated with FCD in Singaporean Chinese, with the allele C conferring a 2.57-fold of increased risk [24]. However, this SNP had not been included in other studies including a recent report in Chinese [23]. Its role in this population has yet to be confirmed. Thalamuthu et al. and Baratz et al. respectively performed haplotype association tests between individual SNPs across the entire *TCF4* gene and the associations were similar [16], [23]. If rs17089887 can be confirmed as a genuine FCD-associated SNP, then there could be another SNP in or near the *TCF4* gene in linkage disequilibrium with both rs613872 and rs17089887 that may be responsible for the association signals detected on these two SNPs.

FCD has reportedly lower prevalence among Chinese than Caucasians, and it accounts for only 3%–4.5% of cases requiring penetrating keratoplasty [31]–[33]. The low occurrence may be due to the non-polymorphic *TCF4* rs613872 in Chinese as shown in our meta-analysis. Furthermore, according to the database of the Human Genome Diversity Project [34], the risk allele G of *TCF4* rs613872 is rare or nonexistent in populations from eastern Asia, Africa, and central and southern America but is more frequent in European, Middle Eastern and southern Asian populations. In order to determine the association of *TCF4* rs613872 with FCD, further studies involving larger cohorts are thus needed to correlate the prevalence of FCD and the frequencies of *TCF4* polymorphisms in other regions of China and Asia.

The *TCF4* gene is located on 18q21.1, encoding the E2-2 protein, a member of the ubiquitously expressed class I basic helix-loop-helix (bHLH) transcription factors which are necessary in cell growth and differentiation [35]. There is expression of E2-2 in the developing corneal epithelium [17]. TCF4 can induce epithelial-mesenchymal transition (EMT) in epithelial cells, with a loss of the epithelial morphology, distinctive epithelial markers, and a gain or reorganization of mesenchymal markers, through an indirect E-cadherin repression and induction of downstream EMT regulators ZEB1 [36]–[38]. Defective EMT has been proposed as a potential pathway for FCD by impairing the mobilization of stem cells to repair corneal damage [5], [17], [39]. The TCF4 induced ZEB1 was also shown to be related to tumor invasiveness due to its effects in EMT [40]. ZEB1 was also shown to regulate type I collagen expression and is thus critical for the maintenance of an endothelial phenotype [41], [42]. *TCF4* rs613872 is within the chromatin immunoprecipitation sequence (ChIP-seq)-purported binding site for two transcription factors: *Ini1 (SMARCB1)* and *BRG1 (SMARCA4)* [43]. These two factors are components of the SWI/SNF chromatin remodeling complex involved in transcriptional regulation [25], [26]. It has been postulated that variation over rs613872 would affect the spatiotemporal expression of *TCF4* through *Ini1 (SMARCB1)* and *BRG1 (SMARCA4)* and hence its targets [19]. Though more evidence is needed to support this linkage, the association in *TCF4* rs613872 played a significant role in identifying the potential causative genes and possibly future biomarkers for FCD.

This systematic review and meta-analysis is an overview of the published genetic studies on *TCF4* and *PTPRG* in FCD. The study has also revealed the limitations in the current FCD genetic studies. In particular, only a limited number of SNPs were found for this meta-analysis, and the number was even smaller in the Chinese populations making false negative errors likely. The small number of studies with small number of study participants in Chinese and Asian populations and non-polymorphism in the relevant SNPs had limited the generalizability of our results and introduced heterogeneity in the data. Further studies on *TCF4* in FCD among different Chinese and Asian populations are needed to confirm the role of this gene. Besides, the homogeneity of the ethnical background in different Caucasian study cohorts may have played a role giving rise to subtle difference in the prevalence and severity of FCD which have not been accounted for in this study. It would be of higher impact and generalizability if more details and various ethnicities in relation to FCD were reported. Also, although a comprehensive search has been employed with different strategies to identify all published studies on *TCF4* and *PRPRG*, relevant studies not meeting the search criteria can be missed. There may be useful results from association studies reported in abstracts, conferences and non-English journals. Thus, there can be selection and publication bias as a result of disproportionate exclusion of negative data, although we detected no significant bias by the Egger's test in the present study. Finally, important covariates like gender, age, severity of FCD, or potential gene-gene and gene-environment interactions could not be included in our analysis because raw allelic and genotypic data were not available. However, alleles are unlikely to display opposite effects based on different covariate. The association between FCD and variants in *TCF4* and *PTPRG* as detected in this study should therefore be genuine.

In summary, results of this meta-analysis confirm 5 SNPs in *TCF4*, with rs613872 having the strongest effect, to be genetic susceptibility factors for FCD in Caucasians but not Chinese populations. To enhance our understanding of the mechanism of FCD, re-sequencing studies and biological functional studies in multiple ethnic groups are required to ascertain FCD-associated gene variants and to determine their pathophysiological effects.

## Supporting Information

Appendix S1 **Lists of included studies.**
(DOC)

Appendix S2 **Lists of excluded studies with reason.**
(DOC)

Checklist S1 **PRISMA checklist.**
(DOC)
