# Supplementary material for: Association of Common Variants in TCF4 and PTPRG with Fuchs' Corneal Dystrophy: A Systematic Review and Meta-Analysis
Source: PLoS One. 2014 Oct 9;9(10):e109142. doi: 10.1371/journal.pone.0109142 (PMC4192317; doi:10.1371/journal.pone.0109142)
Supplement: Appendix S1 — Lists of included studies. (DOC) [file pone.0109142.s001.doc]

Appendix S1 Lists of included studies

[1-8]

1. Igo, R.P., Jr., et al., *Differing roles for TCF4 and COL8A2 in central corneal thickness and fuchs endothelial corneal dystrophy.* PLoS One, 2012. **7**(10): p. e46742.

2. Baratz, K.H., et al., *E2-2 protein and Fuchs's corneal dystrophy.* N Engl J Med, 2010. **363**(11): p. 1016-24.

3. Wang, K.J., et al., *Association of Transcription Factor 4 (TCF4) and Protein Tyrosine Phosphatase, Receptor Type G (PTPRG) with Corneal Dystrophies in Southern Chinese.* Ophthalmic Genet, 2013.

4. Stamler, J.F., et al., *Confirmation of the association between the TCF4 risk allele and Fuchs endothelial corneal dystrophy in patients from the Midwestern United States.* Ophthalmic Genet, 2013. **34**(1-2): p. 32-4.

5. Kuot, A., et al., *Association of TCF4 and CLU polymorphisms with Fuchs' endothelial dystrophy and implication of CLU and TGFBI proteins in the disease process.* Eur J Hum Genet, 2012. **20**(6): p. 632-8.

6. Thalamuthu, A., et al., *Association of TCF4 gene polymorphisms with Fuchs' corneal dystrophy in the Chinese.* Invest Ophthalmol Vis Sci, 2011. **52**(8): p. 5573-8.

7. Li, Y.J., et al., *Replication of TCF4 through Association and Linkage Studies in Late-Onset Fuchs Endothelial Corneal Dystrophy.* Plos One, 2011. **6**(4).

8. Riazuddin, S.A., et al., *Replication of the TCF4 intronic variant in late-onset Fuchs corneal dystrophy and evidence of independence from the FCD2 locus.* Invest Ophthalmol Vis Sci, 2011. **52**(5): p. 2825-9.
