# Supplementary material for: Association of Common Variants in TCF4 and PTPRG with Fuchs' Corneal Dystrophy: A Systematic Review and Meta-Analysis
Source: PLoS One. 2014 Oct 9;9(10):e109142. doi: 10.1371/journal.pone.0109142 (PMC4192317; doi:10.1371/journal.pone.0109142)
Supplement: Appendix S2 — Lists of excluded studies with reason. (DOC) [file pone.0109142.s002.doc]

Appendix S2 Lists of excluded studies with reason

Article [1] studied mutation in the *TCF8* gene rather than *TCF4* in FCD subjects.

Article [2] studied on family rather population and on other gene *PITX2*.

Article [3] studied on normal participants rather than patients with Fuchs corneal dystrophy.

Article [4] concerned with trinucleotide repeat but not SNP in *TCF4.*

Article [5] was an editorial from N Engl J Med.

Article [6] was a review and excluded due to its nature.

Article [7] was a review and excluded due to its nature.

Paper [8] was an abstract of a poster and data are insufficient for analysis.

1. Riazuddin, S.A., et al., *Missense mutations in TCF8 cause late-onset Fuchs corneal dystrophy and interact with FCD4 on chromosome 9p.* Am J Hum Genet, 2010. **86**(1): p. 45-53.

2. Kniestedt, C., et al., *A novel PITX2 mutation and a polymorphism in a 5-generation family with Axenfeld-Rieger anomaly and coexisting Fuchs' endothelial dystrophy.* Ophthalmology, 2006. **113**(10): p. 1791 e1-8.

3. Mackey, D.A., et al., *Role of the TCF4 gene intronic variant in normal variation of corneal endothelium.* Cornea, 2012. **31**(2): p. 162-6.

4. Wieben, E.D., et al., *A common trinucleotide repeat expansion within the transcription factor 4 (TCF4, E2-2) gene predicts Fuchs corneal dystrophy.* PLoS One, 2012. **7**(11): p. e49083.

5. Wright, A.F. and B. Dhillon, *Major progress in Fuchs's corneal dystrophy.* N Engl J Med, 2010. **363**(11): p. 1072-5.

6. Iliff, B.W., S.A. Riazuddin, and J.D. Gottsch, *The genetics of Fuchs' corneal dystrophy.* Expert Rev Ophthalmol, 2012. **7**(4): p. 363-375.

7. Navarrete, K., et al., *TCF4 (e2-2; ITF2): a schizophrenia-associated gene with pleiotropic effects on human disease.* Am J Med Genet B Neuropsychiatr Genet, 2013. **162B**(1): p. 1-16.

8. Lizarraga, E.P.L., et al., *Association between Fuchs’s Corneal Dystrophy and a Single Nucleotide Polymorphism on Chromosome 18 in Mexican Population.* Clinical and Experimental Ophthalmology, 2012 40 (Suppl 1): 63-118 . Conference: 44th Annual Scientific Congress of the Royal Australian and New Zealand College of Ophthalmologists,
